# Expression and Characterization of *Drosophila Signal Peptide Peptidase-Like* (*sppL*), a Gene That Encodes an Intramembrane Protease

**DOI:** 10.1371/journal.pone.0033827

**Published:** 2012-03-16

**Authors:** David J. Casso, Songmei Liu, Brian Biehs, Thomas B. Kornberg

**Affiliations:** 1 Department of Biochemistry and Biophysics, University of California San Francisco, San Francisco, California, United States of America; 2 Cardiovascular Research Institute, University of California San Francisco, San Francisco, California, United States of America; Consejo Superior de Investigaciones Cientificas, Spain

## Abstract

Intramembrane proteases of the Signal Peptide Peptidase (SPP) family play important roles in developmental, metabolic and signaling pathways. Although vertebrates have one SPP and four SPP-like (SPPL) genes, we found that insect genomes encode one Spp and one SppL. Characterization of the *Drosophila sppL* gene revealed that the predicted SppL protein is a highly conserved structural homolog of the vertebrate SPPL3 proteases, with a predicted nine-transmembrane topology, an active site containing aspartyl residues within a transmembrane region, and a carboxy-terminal PAL domain. SppL protein localized to both the Golgi and ER. Whereas *spp* is an essential gene that is required during early larval stages and whereas *spp* loss-of-function reduced the unfolded protein response (UPR), *sppL* loss of function had no apparent phenotype. This was unexpected given that genetic knockdown phenotypes in other organisms suggested significant roles for Spp-related proteases.

## Introduction

Transmembrane segments of integral membrane proteins can be cleaved by Intramembrane Cleaving Proteases (I-CLiPs; reviewed in) [1]. These integral membrane proteins are remarkable enzymes, with catalytic sites situated within the lipid bilayer. Known I-CLiPs have been categorized into four families: γ-secretase aspartyl proteases, rhomboid serine proteases, Site 2 Proteases (S2P), and signal peptide peptidase (SPP) aspartyl proteases. I-CLiPs carry out essential steps in metabolic and cell signaling pathways, including activation of Notch by Presenilin, the aspartyl protease in the γ-secretase complex [2], [3], [4], cleavage and release of the *Drosophila* EGF-like proteins by Rhomboids [5], and cleavage and activation of SREBP by Site-2 Protease (S2P) [6]. Mammalian SPP was first identified as an enzymatic activity that proteolyzes signal peptides generated by proteolysis in the endoplasmic reticulum (ER) [7], [8]. Its characterization has revealed that in addition to a role in housekeeping functions such as cleansing the membrane of signal peptides, it also cleaves substrates to release bioactive peptides from lipid bilayers. Substrates for SPP include HLA-E [9], hepatitis C virus polyprotein [10], preprolactin [11], and class I MHC heavy chains in cytomegalovirus infected cells [12]. The activities of *Drosophila* Spp are less well characterized, but a recent report identified Crumbs, a transmembrane protein controlling cell polarity and morphogenesis that has an unusually long signal peptide, as a target substrate [13].

Putative SPP homologs (“SPP-like” proteases (SPPLs)) have been identified in the genomes of mammals, amphibians, fish, insects, and nematodes, and related sequences have been found in rice, corn and *Arabidopsis* [8], [14]. Like SPPs, these proteins are characterized by a nine-transmembrane topology, an aspartyl diad (YD and GXGD) in the presumptive catalytic site situated within two transmembrane domains, and a PAL motif of unknown function near the carboxy terminus. Vertebrate genomes encode five SPP family members: SPP itself, and related proteins that have been named, SPPL2a/b/c and SPPL3. Fungal genomes also encode a fifth member, SPPL4. The SPP, SPPL2a/b/c and SPPL3 proteins all appear to have the same relative orientation, placing their catalytic sites in a similar manner within the membrane. This conserved orientation is consistent with the idea that all of these family members cleave type 2 transmembrane proteins by a similar process [15]. To date, target substrates have been identified for only the SPPL2 enzymes. These substrates are TNF-α, Bri2, and FasL [16], [17], [18], [19].

In addition to the biochemical approaches that have been taken to investigate the functions of SPP proteases, genetic studies have been carried out in *C. elegans*, *D. rerio* and *D. melanogaster* that have suggested several types of essential roles for SPP. RNAi knockdown of *C. elegans IMP-2* (*spp*) caused embryonic lethality, abnormal larval molting, adult egg production defects and sterility [20]. In *D. rerio*, knockdown phenotypes for *spp* and *sppl3* included neural lethality, and knockdown of *sppl2b* caused vasculature and blood abnormalities [21]. Reduction of *spp* function in *A. thaliana* compromised pollen formation [22].

We previously characterized the expression and genetics of the *Drosophila spp* gene [23]. Expression of *spp* was first detected in germ band extended embryos, and was present at higher levels in the proventriculus, salivary glands, and trachea of late embryos. The Spp protein localized to the ER. Loss-of-function alleles of *Drosophila spp* were isolated and found to have larval lethal phenotypes and defective tracheal development. Here, we report that the Spp family is conserved in eighteen insect genomes, each having one Spp and one SppL ortholog. We describe a genetic characterization of the sole *Drosophila* SppL-encoding gene, *sppL* (*CG17370*). We found that *sppL* is expressed broadly during early embryogenesis, and that its expression is elevated in mesodermal and midgut primordia in later embryos before waning to undetectable levels in late embryos. And in contrast to Spp, we found that SppL localizes to both the ER and Golgi. Finally, we describe the generation of null alleles of *sppL* and their characterization. Unexpectedly and in contrast to *spp* mutants, *sppL* loss of function has no apparent consequence on development or lifespan.

## Methods

### Cloning and expression of *sppL*

The *sppL* (*CG17370*, FBgn0039381) open reading frame was amplified from a 3–12 hour embryonic cDNA library [24] by PCR, using Vent DNA polymerase (New England Biolabs) and oligonucleotides oEcoRI-sppL-M-s (CCGGAATTCATGTCGCACGGTGGAGCC) and oXhoSppL-a (AAACTCGAGTCAGACTTCCAGTTGTTTTGATGG). The resulting PCR product was first subcloned into pCR2.1 (Invitrogen), creating *pCR2.1-sppL* and subsequently into pUAST [25], creating *pUAS-sppL*. Sequencing confirmed an exact match with the Genbank *CG17370* sequence.

*pUAS-myc-sppL* in which a single amino-terminal myc tag was fused in-frame with the sppL initiating methionine was created using oligos oBgl2mycSPPL-s (AAAGATCTATGGAACAAAAACTTATTTCTGAAGAAGACCTGATGTCGCACGGTGGAGCCGGTGGCGG) and oXhoSppL-a. S2 cells were grown in Shields and Sang M3 media supplemented with 10% heat-inactivated fetal bovine serum, and co-transfected with *pUAS-myc-sppL* and *pA5c-GAL4* [26] using Effectene (Qiagen). One of two marker plasmids was also included in each transfection: *pA5cGG105* expressing a fusion of Calreticulin (Crc), GFP and the “KDEL” ER retention signal, which marks the ER only; or *pA5cGG112* expression a fusion of KDEL Receptor (KdelR) with GFP, which marks the ER and Golgi. Imaging these cells was done as previously described [23].

### *In situ* hybridization

*In situ* hybridizations were carried out as previously described [27] with a 1.2 kb anti-sense digoxygenin-labeled riboprobe for the entire *sppL* protein coding sequence. Embryos were from an overnight collection of *y w* flies.

### Generation of *sppL* mutants

Flies carrying the *P{lacW}l(3)SH116^SH116^* (FBal0143368) element [28] were obtained from the Szeged Drosophila Stock Center. For simplicity, we refer to this element as *P{lacW}sppL^sh116^*. The position and orientation of *P{lacW}sppL^sh116^* in the large intron of *sppL* were determined by amplification and sequencing of the flanking genomic DNA. DNA was amplified from both sides of the *P{lacW}sppL^sh116^* element with the following primer pairs: oEP3Pi (GAGTTAATTCAAACCCCACGGACATGCTAAGGG)+osppL-2000s (CGGCGGTGCTAATGTAGCGCATTTCACTG); and oEP5Pi (CTGACCTTTTGCAGGTGCAGCCTTCCACTGCG)+osppL-4000a (GTAATGAAAATAAAACTCAGAAACTGCGG). Pools of genomic DNA from progeny were generated after imprecise excision of *P{lacW}sppL^sh116^*, and products of PCR amplification using at least four primer pair combinations were tested. The sense primer on the left of the P element insertion site osppL-2000s corresponds to sequence between the *P{lacW}sppL^sh116^* insertion site and exon N2. The four antisense primers to the right of the P element are separated by approximately 1 kb intervals and correspond to sequences: within the large intron (osppL-4000a); exon C1 (osppL-5000a, CATTTCGCTTCTTCTGCTCCCGCTCGCGG); within exon C4 (osppL-6000a, CAATGCCACCCAGATGCAACTTTCTGGCC); and the intergenic sequence between *sppL* and *Lnk* (osppL-7250a, GTTTGCAACGAACACATGCATTTTGGC). Genomic DNA was prepared from adult flies as described in [29].

*Df(3R)sppL* was created by recombination between *FRT* insertions *PBac{RB}CG17370^e00372^* (FBti0047087) and *PBac{XP}Lnk^d07478^* (FBti0042888) [30] which flank the *sppL* gene (according to) [31]. This deletion was verified both by amplification of DNA between the two *PBac* elements and by failure to amplify the *sppL* gene from the *Df(3R)sppL* genomic DNA.

### Spp and SppL homology

*D. melanogaster* Spp and SppL protein sequences were used as queries for NCBI BLASTP [32]. Each sequence was used to identify related proteins both from databases of reference proteins (refseq_protein) and non-redundant protein sequences (nr) from each of the species listed in Table 1. Orthologs were identified as sequences with high amino acid identity throughout and BLASTP scores higher than 470; BLASTP scores comparing Spp to SppL sequences were between 100 and 150. Percent identities (Table 1) were calculated using CLUSTAL W [33]. The phylogram was created using CLUSTAL W2 [34] and TreeVector [35] by comparing sequences for insect Spp and SppL orthologs listed in Table 1 and human SPPSPPL2a (NP_116191), SPPL2b (NP_694533), SPPL2c (NP_787078), and SPPL3. Putative transmembrane domains were identified using the “TMHMM” and HMMTOP algorithms [36], [37] and by similarity to the model proposed by Friedmann *et al.* [15].

**Table 1:** Orthologs of *D. melanogaster* Spp and SppL proteins.

Species	Spp ortholog	Identity with		SppL ortholog	Identity with	
		*Dm* Spp	*Dm* SppL		*Dm* Spp	*Dm* SppL
*D. yakuba*	GE16062	99%	21%	GE23625	23%	98%
*D. erecta*	GG24644	99%	21%	GG11430	23%	98%
*D. simulans*	GD22952	94%	20%	GD21240	23%	98%
*D. sechellia*	GM16661	96%	20%	GM10271	23%	98%
*D. ananassae*	GF24718	95%	21%	GF16778	22%	91%
*D. grimshawi*	GH10510	90%	20%	GH22306	22%	90%
*D. mojavensis*	GI18028	90%	19%	GI22975	23%	90%
*D. willistoni*	GK14664	87%	21%	GK12863	22%	90%
*D. persimilis*	GL19257	91%	20%	GL21795	22%	90%
*D. pseudoobscura*	GA11227	91%	20%	GA14486	23%	90%
*D. virilis*	GJ19708	89%	20%	GJ22735	13*%	57*%
*A. aegypti*	XP_001655809	67%	22%	XP_001648511	22%	79%
*C. quinquefasciatus*	XP_001842495	66%	23%	XP_001861816	22%	79%
*A. gambiae*	AGAP008838	60%	23%	AGAP003207	21%	79%
*N. vitripennis*	XP_001600867	62%	21%	XP_001603590	21%	71%
*T. castaneum*	XP_967836	62%	22%	XP_973970	22%	71%
*A. mellifera*	XP_393360	59%	22%	XP_393189	22%	70%
*H. sapiens*	NP_110416	58%	21%	NP_620584	20%	59%

Orthologs of *D. melanogaster* Spp and SppL proteins. Gene names, GenBank accession numbers and percent identity of each sequence with Spp and SppL are shown. The *D. virilis* SppL sequence (GJ22735), appears to be truncated C-terminal to amino acid 280 (an apparent deletion of the two C-terminal transmembrane domains, including the GXGD and PAL domains), resulting in reduced identity scores (asterisks). The relative identity of the GJ22735 *D. virilis* sequence with the N-terminal 280 residues of *D. melanogaster* SppL is 88%.

### Unfolded Protein Response (UPR) assay

Embryos were collected from control flies (*w^1118^*), from two *sppL* lines (*w^1118^*, *sppL^57D^*/*TM3 Kr*-*GFP*, and, *w^1118^*, *Df(3R)sppL^BW1^*/*TM3 Kr*-*GFP*) and from a *spp* line (*w^1118^*, *Df*(*2L*)*lwr^14^ p*(*lwr* ^+^)/*CyO Kr*-*GFP*) and from a double mutant *spp, sppL* line (*w^118^*, *Df*(*2L*)*lwr^14^*, *p*(*lwr* ^+^)/*CyO ActGFP*, *sppL^BW1^*/*TM6B armGFP*) at 25°C. Egg-laying was for 24 hours, and larvae incubated for an additional 2.5 days at room temperature, at which point mutant larvae lacking GFP fluorescence were selected. Control and mutant larvae were cut in half longitudinally, turned inside out to expose internal tissues, and incubated at 25°C in Shields and Sang M3 Insect Medium supplemented with 10% heat-inactivated fetal bovine serum and penicillin-streptomycin. To induce the UPR, DTT (5 mM) was added to induce the ER stress response. To detect UPR-induced alternative splicing of the *xbp1* transcript, total RNA was prepared after two hours of incubation in media using ZR RNA MicroPrep kit (Zymo) followed by treatment with DNase. cDNA was synthesized from 0.15 µg RNA using a High Capacity RNA-cDNA kit (Applied Biosystems). Thirty cycles of PCR amplification using Vent DNA Polymerase (New England Biolabs) with *XbpI* (*CG9415*, FBgn0021872) primers XBP-F (TCAGCCAATCCAACGCCAG) and XBP-R (CTGTTGTATACCCTGCGGCAG) were carried out using a 60°C annealing step. Products of 100 and 77 base pairs were resolved on 2% Omnipur low melting agarose (EM Scientific) gels, with the smaller band indicative of the UPR. Relative band intensities were measured using ImageJ.

### Life span determination

Ten virgin flies were placed into vials containing standard cornmeal/yeast/agar medium. A census of each vial was taken every seven days, and the surviving flies transferred to a fresh vial until all the flies had died.

## Results

### The SppL protein

**Figure 1:**
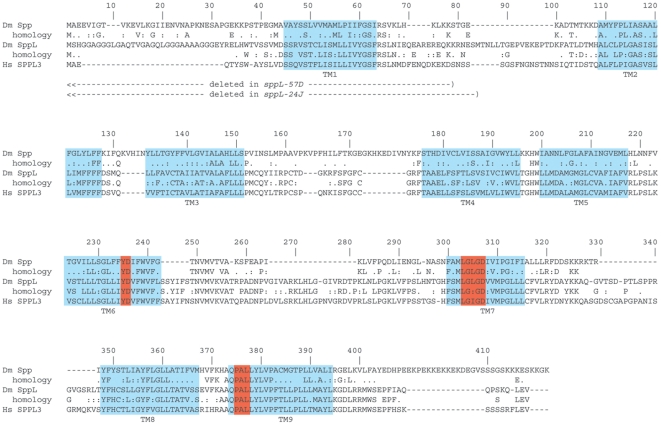
Sequence similarity of the SppL protein to *D. melanogaster* Spp and human SPPL3. Three sequences are shown: *Drosophila* Spp (Dm Spp), *Drosophila* SppL (Dm SppL), and human SPPL3 (Hs SPPL3). Homologies between Dm Spp and Dm SppL, and between presumptive Dm SppL and Hs SPPL3 are indicated: for identity, by a letter; or for similarity, by a colon. Predicted transmembrane domains are highlighted in blue boxes and numbered TM1-TM9. The catalytic regions including the aspartyl diad and PAL motif are shown in red boxes. Dashed lines (—) indicate the extent of the *sppL^24J^* and *sppL^57D^* deletions.

**Figure 2:**
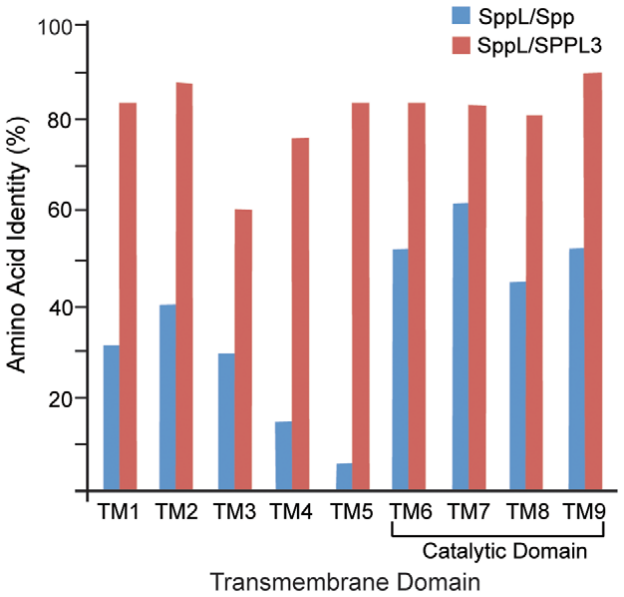
Sequence comparisons of Spp and SppL proteins. Pair-wise comparisons of amino acid identity (%) are plotted for each of the nine predicted transmembrane (TM) domains. Comparisons between *Drosophila* SppL and *Drosophila* Spp are in blue; comparisons of *Drosophila* SppL and human SPPL3 are in red. Whereas strong identity exists between *Drosophila* SppL and human SPPL3 in all transmembrane domains (red), the region of strong identity between Spp and SppL (blue) is limited to the C-terminal four transmembrane domains (TM6-TM9) that include the catalytic domains [47].

In 2002, a search of sequence databases identified the I-CLiP family of presenilin homologs [14] that includes *Drosophila* CG11840 (spp) [23] and CG17370. We have investigated the CG17370 sequence and here show that it encodes a SPPL homolog. *CG17370* is predicted to encode two proteins (417 and 422 residues) that could be derived by alternative splicing. These proteins have limited similarity to the 390 residue *Drosophila* Spp (24% identity with the 417 residue form of CG17370). However, several key features and regions in these proteins suggest their functional homology. Both Spp and CG17370 proteins have nine predicted transmembrane helices (Fig. 1), of which the four C-terminal helices that include the presumed catalytic domain have significant sequence homology (55% identity in this region; Figs. 1, 2). Sequence similarity is particularly high in the immediate vicinity of the catalytic YD and GXGD aspartyl diad, as well as near the distal PAL motif. The putative ER retention motif KKXX found at the carboxy-terminus of SPP proteins [8] is not conserved in CG17370 or SPPL3. The overall relatedness of human SPPL3 to the CG17370 protein (59% overall identity) is significantly higher than is the kinship of *Drosophila* Spp and CG17370 (24% identity). All nine predicted transmembrane helices are highly conserved between human SPPL3 and *Drosophila* CG17370 (80% identity; Fig. 2), and significant sequence similarity is also distributed in the non-transmembrane regions (46% identity). We henceforth refer to the CG17370 protein as SppL.

**Figure 3:**
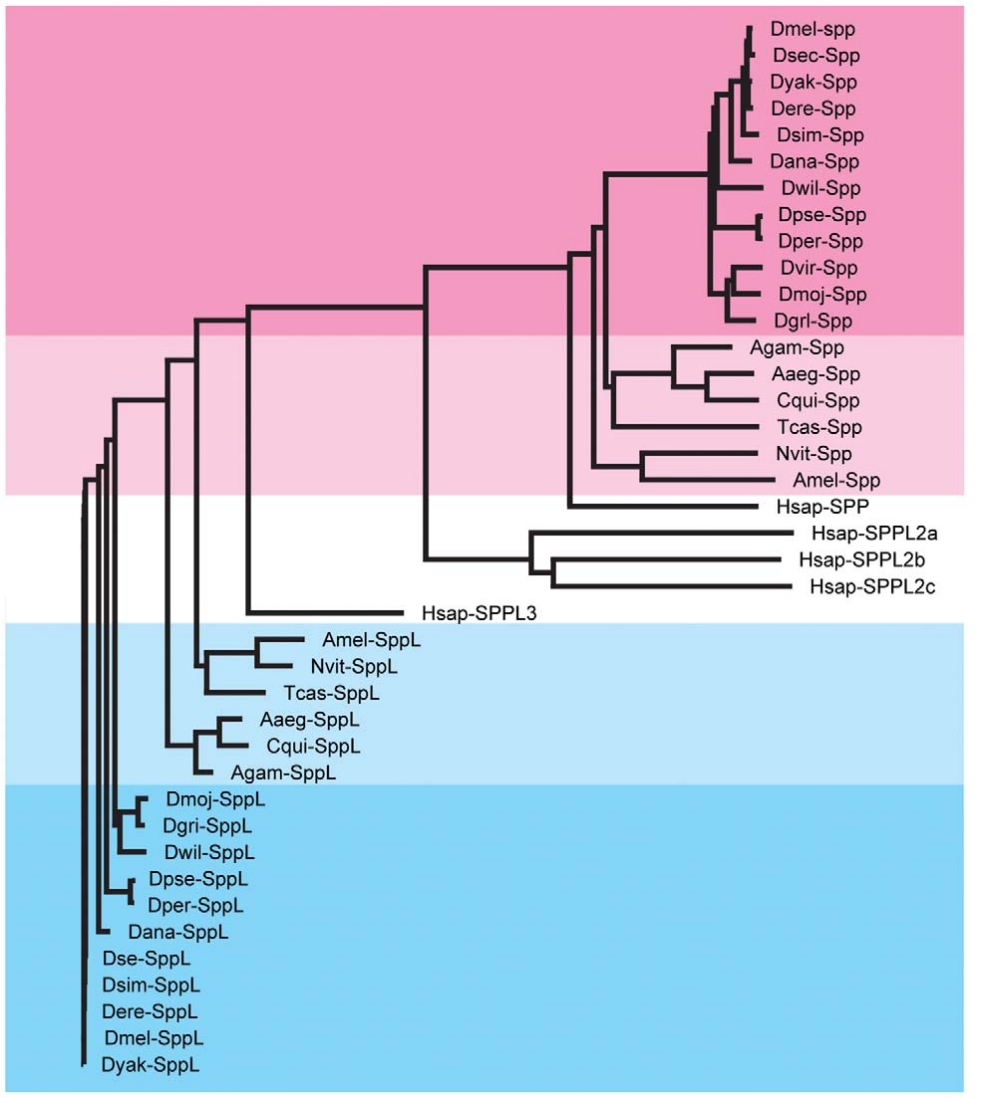
Phylogram of Spp and SppL ortholog sequences. Sequences are marked with an abbreviation for the species (i.e., *D. melanogaster* SppL, Dmel-SppL; see Table 1 for a list of species); accession identifiers for each sequence are in Table 1 or in METHODS. The magenta box groups the *Drosophila* Spp orthologs; light pink, other insect Spp orthologs; white, human SPP, SPPL2a, SPPL2b, SPPL2c, SPPL3; blue, *Drosophila* SppL orthologs; and light blue, other insect SppL orthologs. SppL proteins are more closely related to human SPPL3 than to SPP or SPPL2a, b, or c; and the SppL sequences retain a higher interspecies conservation than Spp sequences. (The apparently truncated sequence of the *D. virilis* SppL ortholog is not included in this analysis.).

The presence of just two members of the Spp family encoded in the genome of *D. melanogaster* contrasts with a larger family of five found in vertebrate genomes. To determine whether the two member Spp family is unique to the species *melanogaster* or is characteristic of the insects, we compared the Spp and SppL sequences from *melanogaster* to the predicted proteomes of ten other *Drosophila* species (*D. ananassae*, *D. erecta*, *D. grimshawi*, *D. mojavensis*, *D. persimilis*, *D. pseudoobscura*, *D. sechellia*, *D. simulans*, *D. willistoni*, and *D. yakuba*), to three species of mosquito (*A. aegypti*, *C. quinquefasciatus*, and *A. gambiae*), to a honeybee (*A. mellifera*), to a wasp (*N. vitripennis*), and to a beetle (*T. castaneum*). BLAST searches revealed that all seventeen genomes encode one Spp and one SppL protein. Similar searches identified all five Spp family members in the human genome. As shown in Table 1, the sequences of the SppL orthologs are strongly conserved between *melanogaster* and the other eleven *Drosophila* species, but the SppL orthologs are all distinct from *melanogaster* Spp. Spp orthologs have been similarly conserved and are distinct from *melanogaster* SppL. Conservation is also significant for the Spp and SppL orthologs in the other six insect genome sequences we analyzed. Comparison of *D. melanogaster* SppL to the *H. sapiens* sequences SPPL2a, SPPL2b, SPPL2c and SPPL3 revealed that only SPPL3 had significant sequence conservation (12%, 16%, 15% and 59% identity, respectively). And conservation of *H. sapiens* SPP and *melanogaster* Spp is highly significant (58%) while conservation of *H. sapiens* SPP and *melanogaster* SppL is less (29%). Note that the sequence conservation of *D. melanogaster* SppL and *H. sapiens* SPPL3 (59% identity) is almost as great as the similarity of *D. melanogaster* SppL to orthologs of non-Drosophila insects (70–79%) and far greater than the similarity between *D. melanogaster* SppL and Spp (24%). The sequence conservation of *H. sapiens* SPPL3 with SPPL2a/b/c is similarly low (13%, 16% and 16%, respectively). These data suggest that insects encode single species of Spp and SppL proteins, that *H. sapiens* SPPL3 is an ortholog of the insect SppL proteins, and that *H. sapiens* SPP is an ortholog of the insect Spp proteins. These relationships are apparent in the phylogram illustrated in Figure 3.

### *sppL* expression and SppL subcellular localization

**Figure 4:**
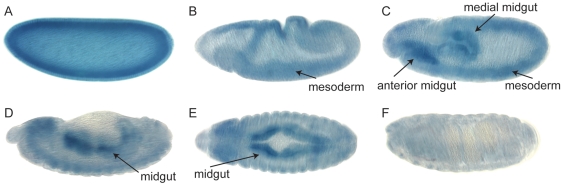
Expression of *sppL* in *Drosophila* embryos. (A) A uniform distribution of *sppL* transcripts was detected near the surface of embryos by *in situ* hybridization at the cellular blastoderm stage. (B) At early germ band extension (stage 7), mesodermal expression is apparent. (C) At late germ band extension (stage 9), strong expression of *sppL* is seen in the developing midgut. (D) At germ band retraction (stage 12) and (E) dorsal closure (stage 14), midgut expression remains strong, while mesodermal expression is beginning to fade. (F) By late embryogenesis (stage 16), expression of *sppL* is no longer detectable.

To determine if *Drosophila sppL* is expressed, we probed embryos and larvae for transcripts by *in situ* hybridization. In embryos, we detected *sppL* transcripts that were uniformly distributed at cellular blastoderm (Fig. 4A). During gastrulation, expression in the mesoderm was prominent during early germ band extension (Fig. 4B), and was more pronounced at full germ band extension stages in the anterior and medial portions of the midgut (Fig. 4C). Expression continued to be strong in the midgut after germ band retraction, while expression in the mesoderm diminished (Fig. 4D, E). By late embryogenesis, expression of *sppL* was no longer detected (Fig. 4F). Although we did not detect expression in larval imaginal tissues (data not shown), transcriptional profiling reported by modENCODE identifies expression at all stages [38]. The expression program of *sppL* contrasts with that of *spp* [23]. *spp* expression was not detected in blastoderm stage embryos, but was detected during later embryo stages and in imaginal discs [23]. Expression in the developing trachea of embryos was consistent with the presence of incomplete tracheal air filling in *spp* mutants [23].

**Figure 5:**
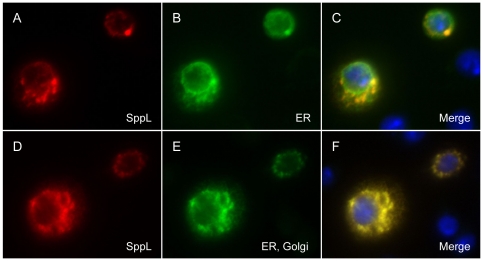
SppL localization to the Golgi and ER. S2 cells were transfected two express either (A–C) MYC-SppL and Crc-GFP-KDEL marking ER, or (D–F) MYC-SppL and KDEL Receptor-GFP marking Golgi and ER. (C, F) In the merged images, MYC-SppL is in red the GFP fusion proteins are in green. Hoechst staining of nuclei is included in blue (note that only two cells in each frame were transfected). While some colocalization of SppL and the ER marker can be seen in C, extensive colocalization with the ER and Golgi marker is evident in F.

We examined the subcellular localization of SppL and compared it with that of Spp. Spp protein was found primarily in a strong perinuclear ring and reticular pattern that is consistent with the morphology of the ER, and it co-localized with the ER marker Calreticulin-GFP-KDEL. In order to detect SppL protein, we engineered a MYC tag at the amino terminus of SppL. When this protein was expressed in *Drosophila* S2 cells, we detected a punctate staining pattern accompanied by a weak perinuclear ring. This pattern contrasts with the ring of expression and lacy reticular staining of Spp. While there was some co-localization of SppL and Calreticulin-GFP-KDEL, the two patterns were distinct (Fig. 5A–C). SppL did co-localize almost perfectly with KDEL receptor-GFP, suggesting that SppL resides in both the ER and Golgi (Fig. 5D–F). The intracellular distribution of SppL is similar to that of human SPPL3, which localizes predominantly to the Golgi [19]. Possibly relevant are sequences in Spp and SppL that may target them to the secretory pathway. However, whereas Spp has a C-terminal KKXX motif that putatively targets it for ER retention, intracellular localization of Spp was unchanged when a C-terminal Myc sequence tag was fused downstream of this sequence [23].

### *sppL* mutations

**Figure 6:**
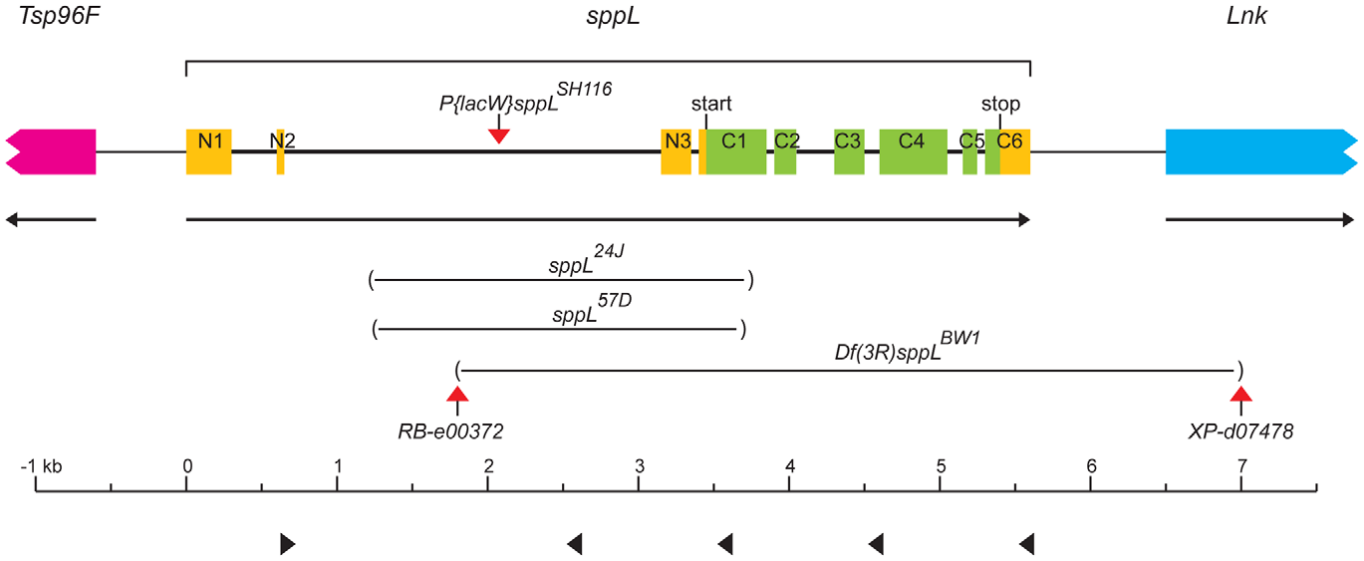
The *sppL* locus. This cartoon of 9.5 kb of chromosome III at cytological band 96F5-6 depicts the *sppL* gene and the ends of the adjacent *Tsp96* (pink) and *Lnk* (blue) genes. Colored boxes indicate the *sppL* exon structure: coding regions (green) and non-coding 5′ and 3′ UTRs (yellow). The predicted “start” and “stop” codons of *sppL* are indicated. Exons N1-N3 are entirely non-coding, while exons C1–C6 contain the *sppL* open reading frame. The insertion sites of transposons *P{lacW}sppL^SH116^* (also known as *P{lacW}l(3)SH116^sh116^*), *PBac{XP}Lnk^d07478^*, and *PBac{RB}CG17370^e00372^* are indicated with red triangles. Imprecise excision of *P{lacW}sppL^SH116^* generated the deletion alleles *sppL^24J^* and *sppL^57D^*. Recombination between the two *PBac* insertions was used to generate deletion *Df(3R)sppL*. The extent of these deletions is indicated within parentheses. The *sppL^57D^* deletion (not shown) is similar to *sppL^24J^*. Black triangles indicate the positions of proximal (▸) and distal (◂) primers used to screen for excision mutants, denoting the positions of the following oligo sites: osppL-2000s, osppL-4000a, osppL-5000a, osppL-6000a, and osppL-7250a.

To assess *sppL* function by loss-of-function genetics, we made *sppL* deletion alleles in two ways. First, we removed a portion of the *sppL* transcription unit by imprecise excision of a P transposon (*P{lacW}sppL^SH116^*). *sppL* is predicted to produce five transcripts that are distinguished by alternate use of three non-coding exons that contribute to the 5′UTRs of all of the *sppL* mRNA species [39; see Fig. 6]. These five transcripts share a large 5′-proximal intron where *P{lacW}sppL^SH116^* has inserted. *P{lacW}sppL^SH116^* was isolated as a lethal in a screen for P element mutations [28]. We determined that recombination of the *P{lacW}sppL^SH116^* chromosome yielded viable transposon-bearing chromosomes, indicating that the lethality of *P{lacW}sppL^SH116^* does not reside with the insertion. We sequenced PCR products amplified with primers that flanked the published insertion site and confirmed its orientation and location 2168 bases downstream of the most 5′ *sppL* start site and within the large *sppL* intron (Fig. 6). *P{lacW}sppL^SH116^* flies were engineered to express P element transposase, and progeny were screened to identify approximately 1000 that lacked the *w^+^* marker of *P{lacW}*. Lines were created from these *w^−^* excisions, and genomic DNA from these lines was then screened in pools of ten using four PCR reactions. The positions of the proximal primer (▸ at 1.6 kb) and four distal primers (◂ at 2.6, 3.6, 4.6, and 5.7 kb) are indicated in Figure 6. Deletions resulting from imprecise excision generated PCR products that were cloned and sequenced. Ten independent deletions within *sppL* ranging in size from 0.8 to 2.5 kb were identified. Deletions *24J* and *57D* were the largest. Proximal to the transposon insertion, they eliminate the branch points for the introns between exons N2-N3 and N2-C1; distally, they remove the translation start, the first transmembrane (e.g. TM1) domain, and part of the loop between TM1 and TM2 (Figs. 1, 6).

Second, a deletion (*Df(3R)sppL*) was created by selecting *w^−^* recombinants between chromosomes carrying *FRT* elements *PBac{RB}CG17370^e00372^* and *PBac{XP}Lnk^d07478^* [30] that flank the *sppL* protein-coding region [31]. *Df(3R)sppL* deleted all *sppL* sequence from a point 5′ of the coding region within the large intron and extends into the neighboring *Lnk* gene (Fig. 6). *Lnk*, which has been implicated in insulin receptor signaling, is not an essential gene [40], [41], [42]. We confirmed the identity of this deletion by PCR analysis, verifying recombination between the *FRT* elements (according to) [31] and the inability to amplify *sppL* sequences from deletion homozygotes (not shown).

The *Df(3R)sppL, sppL^24J^* and *sppL^57D^* alleles are viable, and *Df(3R)sppL* could be maintained as a stock without a balancer chromosome (see Table 2). No morphological abnormalities were apparent in these flies. Whereas *sppL^24J^* and *sppL^57D^* were sickly as homozygotes and were poorly viable, both were viable *in trans* with *Df(3R)sppL* and eclosed with Mendelian frequencies. In addition, *sppL* hemizygotes had similar life spans compared to heterozygous siblings. Female *sppL^24J^*/*Df(3R)sppL* and *sppL^57D^*/*Df(3R)sppL* lived an average of 13.9±2.6 and 11.2±2.4 weeks, respectively, while males of the same genotype lived 13.2±2.7 and 9.9±3.0 weeks. Heterozygous male and female *Df(3R)sppL*/*TM3 Sb^1^* lived 12.6±1.4 and 8.8±2.0 weeks. All these measured lifespans are similar to wild type strains [43], [44], indicating that *sppL* is not an essential gene under the conditions we tested.

**Table 2:** *sppL* stocks and double mutants.

Genotypes	Viability
*sppL^57D^*	viable
*sppL^24J^*	viable
*Df(3R)sppL*	viable
*sppL^57D^*/*Df(3R)sppL*	viable
*sppL^24J^*/*Df(3R)sppL*	viable
*spp^5^*/*Df(2L)lwr^14^*, *p(lwr^+^)*	early larval lethal
*spp^5^*/*Df(2L)lwr^14^*, *p(lwr^+^)*; *sppL^24J^*/*Df(3R)sppL*	early larval lethal
*S2P^1^*	viable
*S2P^1^*; *sppL^24J^*/*Df(3R)sppL*	viable

*sppL* stocks and double mutants. Terminal phenotypes of *sppL* and *SppL* in combination with *spp* and *S2P* are listed.

To investigate whether *sppL* function is redundant to other I-CLiPs, these *sppL* alleles were crossed with *spp* and *S2P* mutants. Whereas loss of *spp* was lethal during early larval development, removal of *sppL* in the haplo-*spp* backgrounds *spp^5^*/+ or *Df(lwr)^14^*, *P(lwr^+^)/+* heterozygotes had no noticeable effect on viability, morphology, or fertility. Lethality of *spp sppL* double mutants occurred during early larval stages, as it did in *spp* mutants, and removal of sppL function did not enhance the *spp* tracheal air-filling defect. There is no confounding maternal effect of *sppL* expression, since *sppL^−^* females were used to generate the double mutant larvae. Over-expression studies were similarly unrevealing. Whereas ectopic expression of *spp* distorts adult wing morphology, no morphological phenotypes were observed in the adult flies after ectopic expression of *sppL* using a variety of strong *GAL4* drivers (e.g., *GMR*, *ptc*, *en*, *T80*) at 29°C. Our experiments therefore did not identify a genetic interaction between *spp* and *sppL*. We also asked if *sppL* interacts genetically with *S2P*, since both of these I-CLiPs are non-essential but might share essential functions. Using the null mutant *S2P^1^*, which can be maintained as a homozygous stock [6], we created double mutants of *S2P^1^* and either *sppL^57D^/Df(3R)sppL* or *sppL^24J^/Df(3R)sppL*. These double mutants were viable, were normal in size and shape, and fertile.

**Figure 7:**
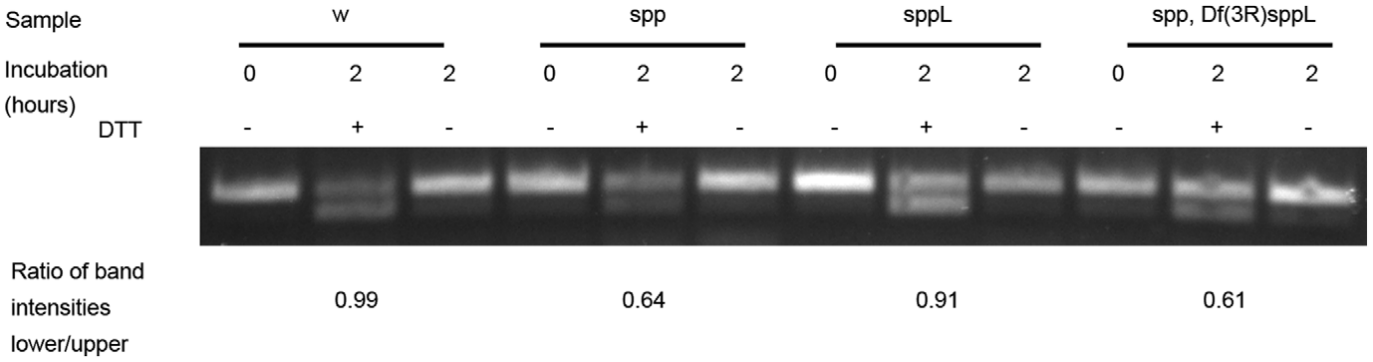
The unfolded protein response in *spp* and *sppL* mutant larvae. A 77 base pair alternative splice product of the *Xbp* I cDNA is made after induction of the UPR by DTT. Genotypes are *w* (*w^1118^), spp (w^118^, Df(2L)lwr^14^ p(lwr^+^)*), *sppL* (*w^118^, Df(3R)sppL*), and spp sppL (*w^118^, Df*(*2L*)*lwr^14^*, *p*(*lwr* ^+^), *sppL^BW1^*). Samples of RNA were prepared from freshly dissected larvae (0 hour) or from larvae incubated in media for 2 hours with or without DTT. Relative intensities of the upper (100 bp) and lower (77 bp) bands in the experimental samples were calculated from the total intensity in each band measured with ImageJ.

The accumulation of misfolded proteins in the ER triggers the unfolded protein response (UPR) [45]. Because the vertebrate SPP protein was been reported to be associated with the enzymes responsible for carrying out ER-associated degradation [46], and because loss of secretory pathway intramembrane proteases might increase uncleaved proteins or peptides in the ER, we examined the UPR in *spp* and *sppL* mutants. Unexpectedly, our assays of the UPR-induced alternative splicing of *XbpI* in control and mutant larvae revealed a decrease of the UPR in the absence of *spp* (Fig. 7). Lack of *sppL* function had no apparent effect on these assays of the UPR.

## Discussion

The presence of *sppL* in the *D. melanogaster* genome is not unique among insects; indeed, BLAST searches of the genome sequences of sixteen other insect species revealed that all include genes that can encode one Spp and one SppL protein (Table 1). BLAST searches identified the five vertebrate Spp family members, but only two were detected in insect genomes. The genomes we queried were from ten other *Drosophila* species (*D. ananassae*, *D. erecta*, *D. grimshawi*, *D. mojavensis*, *D. persimilis*, *D. pseudoobscura*, *D. sechellia*, *D. simulans*, *D. willistoni*, and *D. yakuba*), three mosquito species (*A. aegypti* and *A. gambiae*), honeybee (*A. mellifera*), wasp (*N. vitripennis*) and beetle (*T. castaneum*). In each genome, the SPPL protein retains higher sequence homology to human SPPL3 and *D. melanogaster* SppL than it does to SPP or to the vertebrate SPPL2a/b/c proteins (Fig. 3).

Spp and SppL share overall topology and conserved motifs, including putative aspartyl protease active sites (Figs. 1, 2). Both Spp and SppL proteins purified from bacterial extracts can cleave a model prolactin signal sequence, suggesting that their activities do not depend on protein glycosylation or on other associated proteins [47]. Despite these similarities, Spp and SppL are distinct. Their expression patterns are largely non-overlapping during development, and their subcellular locations differ (Figs. 4, 5). However, because SPP family proteases are thought to have substrate specificity in vivo [13], we cannot yet comment on putative activities of SppL. Over-expression of Spp caused developmental defects such as wing truncations; in contrast, ectopic expression of SppL produced no apparent defects.

Most strikingly, *spp* provides an essential function during development, while *sppL* is not required for viability or patterning. Because knockdown of *Xenopus, C. elegans* and *D. rerio SPPL* genes caused significant developmental abnormalities, we expected to identify an essential role for *Drosophila sppL*, but *sppL* mutants developed without apparent defects, and *spp sppL* double mutants were indistinguishable from *spp* single mutants (Table 2). This contrasts with *Drosophila spp* [23] and with *presenilin*, for which functional disruption in a variety of organisms causes developmental defects due to the failure to activate the Notch signaling pathway (for review, see) [48]. Spp targets type 2 transmembrane segments, and since the putative catalytic sites of SppL and Spp have the same orientation in their respective transmembrane segments 6 and 7, functional redundancy of SppL with either Spp might be expected. Yet despite the absence of an apparent *sppL* mutant phenotype, the strong evolutionary conservation of this gene suggests that SppL might be redundant with another I-CLiP(s) or protease(s), precedents being S2P and the caspase Drice in the *Drosophila* SREBP pathway [49].

A recent report on the toxicity of over-expressed human Huntingtin protein in *Drosophila* indicates that loss-of-function alleles of *spp* and *sppL* reduced Huntingtin-induced motor deficits in mutant flies [50]. Thus, whereas *sppL* loss-of-function alleles do not manifest apparent insufficiency under the standard laboratory conditions, the “sensitized” genetic background in which Huntingtin is over-expressed unmasked a critical role for *sppL* function. Further studies of Huntingtin may be aided by the *sppL* mutants and expression patterns we have described, and such studies may lead to a better understanding of the putative genetic redundancy of *sppL*.

Human SPP may be a component of the ER-associated protein degradation (ERAD) response [46]. Although our assays did not identify a role for *sppL* in the ER stress response, we observed that loss of *spp* decreased the UPR, a result that suggests that Spp activity might facilitate the UPR. Our data does not discriminate between any of the possible mechanisms for this role.

Our findings are reminiscent of genetic studies on the *SPP*-related genes of *C. elegans*. The *C. elegans* genome encodes a single SPP (Imp-2) protein, a closely related SPPL (Imp-1), and a distantly related SPP-like sequence (Imp-1) [14]; the *C. briggsae* genome encodes a comparable cadre of SPP relatives. As with *Drosophila spp* and *sppL* mutants, RNAi directed against *imp-2* caused developmental defects, while RNAi directed against *imp-1* and *imp-3* caused no obvious developmental abnormalities [20]. These data for *Drosophila* and *C. elegans* contrast with genetic studies in zebrafish, in which developmental defects were observed after *spp*, *sppL2a* or *sppL3* were targeted by morpholinos [21]. We suggest that in contrast to the invertebrate proteins, the vertebrate SPP family proteins acquired new essential functions by processes of gene duplication and diversification.
